# Neutrophil activation by *Escherichia coli* isolates from human intestine: effects of bacterial hydroperoxidase activity and surface hydrophobicity

**DOI:** 10.1002/2211-5463.12796

**Published:** 2020-02-05

**Authors:** Mariam Moshkovskaya, Tatyana Vakhrusheva, Daria Rakitina, Julia Baykova, Oleg Panasenko, Lilia Basyreva, Sergey Gusev, Alexander Gusev, Elena Mikhalchik, Natalia Smolina, Gennadiy Dobretsov, Petr Scherbakov, Asfold Parfenov, Nina Fadeeva, Olga Pobeguts, Vadim Govorun

**Affiliations:** ^1^ Federal Research and Clinical Center of Physical‐Chemical Medicine of FMBA Moscow Russia; ^2^ Moscow Clinical Scientific Center Central Scientific Institute of Gastroenterology Moscow Russia

**Keywords:** chemiluminescence, Crohn’s disease, *E. coli*, fluorescent probes, neutrophil activation, peroxidase

## Abstract

Successful colonization of the intestine requires that bacteria interact with the innate immune system and, in particular, neutrophils. Progression of inflammatory bowel diseases (IBD) is associated with alterations in gut microbiota, and dysbiosis in Crohn’s disease (CD) patients is often associated with an expansion of *Escherichia coli*. Here, we investigated the ability of such *E. coli* isolates to avoid neutrophil activation and to utilize reactive oxygen species. Neutrophil activation was detected *in vitro* in normal human blood via luminol chemiluminescence (CL) induced by reactive oxygen and halogen species generated by neutrophils. No significant difference in neutrophil activation *in vitro* was detected between isolates from inflamed (23 isolates) vs healthy intestines (5 isolates), with 10‐fold variation within both groups (2.9–61.2 mV). CL activity of isolates from the same patient differed by 1.5–5 times. Twenty‐four isolates from ileal aspirate, biopsy, and feces of seven patients with CD and one patient with no intestine inflammation were tested for extracellular peroxidase and catalase activity and cell surface hydrophobicity. Average values between patients varied from 26 ± 3 to 73 ± 18 µmol·g^−1^ of air dry weight for peroxidase activity, from 15 ± 2 to 189 ± 56 mmol·g^−1^ of air dry weight for catalase activity, and from 5 ± 3 to 105 ± 9 a.u. for the hydrophobic probe fluorescence. Extracellular peroxidase activity and hydrophobicity of bacterial cell surface correlated negatively with stimulated neutrophil CL. The ability of some isolates to avoid neutrophil activation and to utilize reactive oxygen species may provide a strategy to survive assault by the innate immune system.

## Graphical abstract

The ability of *Escherichia coli* isolates from the intestines of Crohn’s disease (CD) patients to avoid neutrophil activation and to utilize reactive oxygen species was analyzed. Neutrophil activation was detected *in vitro* in normal human blood via luminol chemiluminescence (CL). Extracellular peroxidase activity and hydrophobicity of bacterial cell surface correlated negatively with stimulated neutrophil CL.

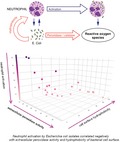

**Abbreviations**
ANS: 1‐anilino‐8‐naphthalenesulfonate
CD: Crohn’s disease
CFU: colony‐forming units
CL: chemiluminescence
DMC: 4‐dimethylaminochalcone
DMEM: Dulbecco’s modified Eagle’s medium
DW: dry weight
FBS: fetal bovine serum
IBD: inflammatory bowel disease
KRB: Krebs–Ringer bicarbonate
OD: optical density
PBS: phosphate‐buffered saline
PMA: phorbol 12‐myristate 13‐acetate
RHS: reactive halogen species
ROS: reactive oxygen species

Progression of inflammatory bowel diseases (IBD) is associated with alteration in gut microbiota. Dysbiosis often observed in CD patients is associated with an expansion of *Escherichia coli* capable of various adaptive reactions 1, 2, 3. One of these reactions is surviving the attack of human immune system. Thus, antibodies to *E. coli* were found in biopsy samples from ulcer, erosion, and granuloma of patients with Crohn’s disease, suggesting the presence of bacteria at the very spot of acute inflammation 4.

It is hard to estimate whether it would be more profitable for bacteria to avoid neutrophil stimulation, or to provoke it. On the one hand, negative correlation between neutrophil‐stimulating capacity and virulence was shown for uropathogenic *E. coli* 5. At the same time, ROS/RHS produced by activated neutrophils at the site of inflammation can enhance damage of gut wall 6, 7, 8 facilitating further bacterial expansion 3.

Upon activation, neutrophils produce reactive oxygen (^•^O_2_ˉ, H_2_O_2_, ^•^OH, etc.) and halogen (HOCl, HOBr, etc.) species (ROS/RHS), which can be detected by chemiluminescence (CL) 9. One of the key products of activated neutrophil, hydrogen peroxide, can be decomposed by bacterial hydrogenases (HPI and HPII) 10, 11, 12, 13. Unlike HPII, HPI shows not only catalase but also peroxidase activity and oxidizes *o*‐dianisidine in the presence of H_2_O_2_ 14, 15. The expression of hydrogenases is regulated by *E. coli* interaction with phagocytes and reorganization of inner and outer cell membrane 16.

Activation of neutrophils could depend on bacterial properties such as hydrophobicity and surface charge of bacterial cell membranes 17, 18, and expression of mannose*‐*sensitive adhesins 19, 20, 21 and of ligands for Toll‐4 receptor of neutrophils 22. The hydrophobicity of bacterial cell envelope can be detected by the affinity of *E. coli* for the fluorescent membrane probes. One of them, amphiphilic 1‐anilino‐8‐naphthalenesulfonate (ANS) 23, does not penetrate the bacterial cell 24 because of its negatively charged sulfo group. Another fluorescent probe used in this study, 4‐dimethylaminochalcone (DMC), is neutral and also sensitive to the hydrophobic sites on bacterial cell surface 25.

The aim of our work was to study the interplay of adaptive mechanisms based on peroxidase/catalase activity, surface hydrophobicity, and neutrophil‐stimulating capacity of *E. coli* from the inflamed gut. Whole blood of healthy donors was used in CL assays of ROS/RHS production in order to exclude effects of patients’ immune status. We previously demonstrated that *E. coli* isolates from the healthy or inflamed human intestine were able to induce CL response in neutrophils in these conditions 26.

## Materials and methods

### Chemicals

Lysogeny broth (LB) medium, Krebs–Ringer bicarbonate (KRB) buffer with CaCl_2_, pH 7.4, phorbol 12‐myristate 13‐acetate (PMA), *o*‐dianisidine, luminol, lucigenin, horseradish peroxidase (HRP), hydrogen peroxide (H_2_O_2_) solution (30 wt, %), potassium iodide (KI), 1‐anilino‐8‐naphthalenesulfonate (ANS), and phosphate‐buffered saline, pH 7.4 (PBS), were purchased from Sigma‐Aldrich (St. Louis, MO, USA). A working solution of H_2_O_2_ was prepared immediately before use by diluting the stock with PBS. Its concentration was controlled by measuring the absorbance at 240 nm (molar extinction coefficient of 43.6 m^−1^·cm^−1^ 27), HCl, and H_3_PO_4_. Tris and NaCl were from Chimmed, Russia. 4‐(Dimethylamino)chalcone (DMC) was kindly provided by Krasowitsky B.M. (Institute of Monocrystals, Kharkov, Ukraine).

### Patients

Crohn’s patient donors of ileum content, biopsy, or feces (*n* = 7, 5 male, two female, age 23–47, median 33), healthy feces donors (*n* = 4, 3 male, one female, age 19–28, median 19.5), and healthy blood donors (*n* = 5, female, age 21–64, median 38) were enrolled in the study and gave written informed consent (Table 1). For one person (male, age 14), the informed content was given by legal representatives.

**Table 1:** Parameters of *Escherichia coli* isolates. CD patients and non‐IBD donors of feces and blood used in the study.

		Patient	Isolate	CL max, Lum, LB	Px, μmol·g^−1^ dry weight	Cat, nmol H_2_O_2_·g^−1^ dry weight	Groups of the isolates according to their CL‐stimulating capacity	*Escherichia coli* genome 29	*Escherichia coli* phylogroup b 29	F (ANS). a.u.	F (DMC). a.u.	Localization 30	Clinical activity 30	Endoscopic activity 31	Type of material for preparation of isolates	*E. coli* DNA copies per ml aspirate/feces^a^
*E. coli* donors	CD	G	G	24 ± 7	36 ± 1.8	77.0 ± 4.1	2	RCE05‐01	D	32.8	26.2	Ileitis–jejunitis	Severe disease	9	Feces	2.1 x 10^9^ stool
		A	A1	2.5 ± 1	51.89 ± 3.2	108.4 ± 5	1	RCE01‐05	A	41 ± 6	105 ± 9	Ileitis	Mild disease	10	Lumen	1.25 x 10^7^ stool
			A2	3 ± 1.2	59.9 ± 2.5	60.8 ± 4.1	1	RCE01‐02	A						Ileum biopsy	
			A3	2.8 ± 1.5	86.4 ± 3.3	86.9 ± 5	1	RCE01‐06	A						Feces	
			A4	3.5 ± 1.3	51.0 ± 2.1	24.4 ± 3.7	1	RCE01‐04	A						Lumen	
		B	B1	42.3 ± 12	28.7 ± 3	16.7 ± 2	3	RCE03‐01	D	24 ± 3	5 ± 3	Ileocolitis	Moderate disease	14	Ileum biopsy	2.8 x 10^5^ aspirate
			B2	61.2 ± 10	22.1 ± 2	14.4 ± 3.2	3	RCE03‐02	D							
		M	M1	14 ± 6	33.7 ± 3.4	14.4 ± 1.6	1	RCE06‐01	B2	30 ± 6	85 ± 12	Ileocolitis	Mild disease	15	Lumen	2 x 10^5^ aspirate
			M2	18 ± 6	30.0 ± 1.8	8.3 ± 2.1	1	RCE06‐02	B2							
			M3	11.8 ± 5	73.8 ± 2.2	33.8 ± 3.7	2	RCE06‐03	B2							
			M4	8 ± 3	43.7 ± 2.5	13.1 ± 2.4	2	RCE06‐04	B2							
			M5	3.2 ± 2	39.7 ± 3.3	11.5 ± 3.5	2	RCE06‐05	B2							
		Z	Z	31 ± 6	20.8 ± 2.8	78.6 ± 5	3	RCE07‐01	A	20.2	25.5	Ileocolitis	Remission	3	Lumen	1.3 x 10^8^ aspirate
		V	V1	9 ± 2.7	20.1 ± 2.4	273.1 ± 3	1	RCE02‐02	B1	21 ± 5	36 ± 8	Ileocolitis	Mild disease	13	Lumen	5 x 10^4^ aspirate
			V2	41.8 ± 14	26.7 ± 1.8	175.8 ± 12	2	RCE02‐01	B1							
			V3	15.1 ± 4	25.8 ± 3.5	150.3 ± 8.1	2	‐	‐							
			V4	17.6 ± 4	38.0 ± 3.2	158.1 ± 6.3	3	RCE02‐03	B1							
		P	P1	18.7 ± 5	18.8 ± 2.6	105.5 ± 15	1	RCE04‐01	A	35 ± 15	33 ± 9	Ileocolitis–perianal	Severe disease	0	Lumen	5.2 x 10^4^ aspirate
			P2	12.2 ± 5	40.9 ± 3.6	130.0 ± 3.5	1	RCE04‐02	A							
			P3	7.2 ± 3	45.5 ± 2.8	170.4 ± 8	2	RCE04‐03	A							
			P4	9.7 ± 3	45.5 ± 3.6	180.0 ± 10	2	RCE04‐04	A							
			P5	20.4 ± 8	24.7 ± 3	125.1 ± 8	2	RCE04‐05	A						Ileum biopsy	
			P6	10.5 ± 5	32.9 ± 2.7	130.1 ± 7	2	RCE04‐06	A							
	Healthy	C	C	59 ± 13	3.2 ± 0.7	29.1 ± 0.6	3			15	16		No IBD	0	Lumen	4 x 10^2^ aspirate
		h‐1	H1	41.6 ± 5									No IBD		Feces	
		h‐2	H2	10 ± 3.2									No IBD		Feces	
		h‐3	H3	58.5 ± 5.1									No IBD		Feces	
		h‐4	H4	5 ± 2.1									No IBD		Feces	
Blood donors		Blood donor 1											No IBD			
		Blood donor 2											No IBD			
		Blood donor 3											No IBD			
		Blood donor 4											No IBD			
		Blood donor 5											No IBD			

Values are given as M ± SD, where M is mean value between the isolates from one patient. SD—standard deviation.

References to the genomes of *E. coli* isolates are given according to Ref. 29

Localization, clinical activity, and endoscopical activity of inflammation were determined according to Ref. 30, 31

a The amount of *E. coli* DNA was measured in feces or aspirate even if some *E. coli* strains for study were sampled from biopsy. From CD samples, only those with *E. coli* DNA content 1 × 10^4^ copies per ml and above were selected for the study

b Phylogroups of *E. coli* isolates were determined from their genomes (sequencing performed in our previous work 29).

The inclusion criteria for CD patients were endoscopically and radiologically diagnosed, and histologically confirmed Crohn’s disease and the content of *E. coli* DNA in the lumen liquid or feces above 1 × 10^4^ copies per mL. The exclusion criteria were signs of indeterminate colitis, infectious diseases, anamnesis of total colectomy, presence of stoma, and recent (less than 2 months) antibiotic treatment. The inclusion criteria for healthy individuals donating blood or feces were no intestinal or autoimmune diseases in anamnesis, no infection diseases, and no recent antibiotic treatment.

Small intestine aspirates and bowel biopsies were obtained during diagnostic endoscopy procedures, which aim was to confirm the IBD diagnosis or to evaluate the state of relapse or remission. For one person (patient C), the diagnostic endoscopy revealed no signs of IBD/inflammation; therefore, this patient was referred as ‘healthy’ in regard to IBD. Stool samples were collected prior to endoscopy for those subjected to it and as a morning stool sampling procedure for healthy volunteers—donors of feces. Samples were stored in sterile containers at +4 °C for up to several hours prior to *E. coli* isolation.

Blood samples from healthy volunteers were obtained by venipuncture using heparin as an anticoagulant. Blood was used for experiments immediately after the draw.

The study methodologies were conformed to the standards set by the Declaration of Helsinki and approved by Ethics committees of Federal Research and Clinical Center of Physical‐Chemical Medicine of FMBA and Moscow Clinical Scientific Center, Central Scientific Institute of Gastroenterology.

### *Escherichia coli* concentration measurement

*Escherichia coli* concentration in ileal and fecal microbiota samples from patients was estimated using SEPTOSKRIN Kit OneStep Strip (Lytech, Moscow, Russia) according to the manufacturer’s protocol. Total DNA from samples was isolated according to CTAB DNA extraction protocol described by Ref. 28 with some modifications: 0.5 mL of material was suspended in 0.5 mL CTAB Buffer, incubated at 60 °C for 30 min, and mixed with 0.5 mL of chloroform. After centrifugation (9000 ***g***, 10 min at 4 °C), the aqueous phase was mixed with an equal volume isopropanol and 0.1 volume 3M NaOAc, pH 5.2, and placed on −20 °C for 1 h. DNA was collected by centrifugation (10 000 ***g*** for 10 min), washed with cold 80% ethanol, and resuspended in 0.2 mL of H_2_O. Total DNA concentration was measured using NanoDrop ND‐100 (Thermo Fisher Scientific, Waltham, MA, USA) and equalized to 200 ng·µL^−1^. *Escherichia coli* DNA amount in the samples was detected by quantity RT‐PCR with specific primers provided by SEPTOSKRIN Kit (Lytech).

Only CD samples with *E. coli* DNA content of 1 x 10^4^ copies per ml of initial sample and above were selected for downstream experiments.

### Bacterial isolates

*Escherichia coli* isolates were achieved as described in Ref. 29. Briefly, aspirates and feces were diluted by 10^6^ in sterile PBS; biopsy fragments were shaken in 0.2 mL of sterile PBS. Aliquots (100 µL) of the resulting liquid were plated on Petri dishes coated with LB agar and incubated overnight at 37 °C. The species affiliation was determined with matrix‐assisted laser desorption/ionization (MALDI) using a mass spectrometer Bruker Microflex combined with the software Biotyper from Bruker Daltonics, Germany.

### Preparation of bacterial cell suspension

*Escherichia coli* cultures were grown on an orbital shaker (150 rpm) at 37 °C by inoculating isolated colony of bacteria into LB medium. Overnight culture of bacteria was collected by centrifugation (3500 ***g***, 10 min) and washed twice with PBS. The washed cells were suspended in PBS, and their optical density (OD) at 540 nm was measured. To estimate the dry weight (DW, mg·mL^−1^), bacterial suspensions were washed with distilled water and dried. The OD of 1.0 corresponded to DW of 0.16 ± 0.03 mg·mL^−1^. Bacterial pellets were stored at −80 °C.

### Adhesion and invasion assay

The assay was performed according to Ref. 32 with slight alterations. Isolates were cultivated on LB (37 °C, 200 RPM) till mid‐log phase. Bacteria were harvested by centrifugation (3500 ***g***, 15 min), and the pellet was washed with PBS and resuspended in PBS. Bacterial suspension (150 μL, OD at 540 = 0.2) was mixed with 5 mL DMEM + 20% fetal bovine serum (FBS). Bacteria in DMEM + FBS (500 μL) were added to CaCo cells grown in 24‐well plate till the monolayer formation (about 500 000 CaCo cells per well). Control bacteria suspension was collected at this point and plated onto LB agar plates at 10^−3^–10^−4^ dilution (control value). The 24‐well plates with CaCo and bacteria were incubated for 3 h at 37 °C. For invasion test, cell suspension was additionally treated with 1 mL of DMEM (20% FBS) with 300 mg·L^−1^ of gentamicin for 1 h at 37 °C. After incubation, the monolayer was gently washed twice with PBS to remove unbound bacteria and cells were removed from the plate by trypsin (200 μL). Cells were lysed by mixing with 0.5% Triton X‐100 in DMEM (20% FBS). The lysates were plated on LB agar at 10^−2^–10^−3^ dilution (5 μL per agar plate). After 24 h of incubation at 37 °C, colony counts were performed to determine colony‐forming units (CFU).

- Adhesion + invasion values were determined as:
- the number of bacteria adhered–invaded per CaCo cell = CFU per agar plate after adhesion*dilution/500 000 (the number of CaCo cells per well);
- % of adhered–invaded bacteria in bacterial suspension = CFU per agar plate after adhesion/CFU per agar plate in control * 100 (considering corresponding dilutions);
- Invasion value was determined as % of adhered–invaded bacteria surviving gentamicin treatment.

### Chemiluminescence measurements of neutrophil activation

Chemiluminescence measurements were performed using a luminometer LKB Wallac 1251 according to the method described by Ref. 33 with modifications 26. Chemiluminescence was tracked at 37 °C under continuous agitation of a sample, 20 μL of healthy volunteer’s blood and 1 mL of 0.2 mm luminol or lucigenin in KRB solution (pH 7.4) were placed into a luminometer cuvette, and spontaneous chemiluminescence was continuously recorded for one minute. Then, a bacterial suspension in PBS was added to a final concentration of 70 ± 10 μg DW·mL^−1^ (bacteria:neutrophil cell ratio was 4000 CFU/cell), and a chemiluminescence response was recorded. Results were expressed in voltage values (mV) corresponding to maximum CL levels. The responses of donors’ blood to standard activators PMA (100 nm) and opsonized zymosan (0.2 mg·mL^−1^) were 35 ± 19 mV and 80 ± 15 mV, respectively.

To rule out the possible toxic effects of *E. coli* on neutrophils, in a number of experiments, 156 nm PMA was added to the luminometer cuvette at the end of CL analysis, cuvette after the level of *E. coli*‐induced CL had reached its maximum value.

### Extracellular bacterial peroxidase activity

Extracellular bacterial peroxidase activity was estimated by the oxidation of *o*‐dianisidine by bacterial cells in the presence of H_2_O_2_, by method described in Ref. 14 with modifications according to Ref. 34. Two hundred microlitre of 100 mm citrate buffer (pH 5.5) containing 0.32 mm *o*‐dianisidine and 2.3 mm H_2_O_2_ was added to 200 μL of bacterial suspension (3 mg of DW per ml in PBS), and the reaction mixture was incubated for 5 min at ambient temperature. The reaction was stopped by addition of 1 mL of 35% phosphorous acid. The cells were precipitated by centrifugation at 900 g for 20 min, and the OD of supernatants was read at 560 nm. The control probes contained PBS instead of bacterial suspension. The concentration of oxidized *o*‐dianisidine was calculated by using its molar extinction coefficient of 20.040 m ^−1^·cm^−1^ 34. The amount of oxidized *o*‐dianisidine was normalized to a dry‐weight basis. To make sure that the bacterial peroxidase activity is related to the cell wall, bacterial suspensions were incubated with 1% Triton X‐100 for 1 h, and then, the cells were precipitated at 900 ***g*** (30 min), and peroxidase activity was measured in supernatant.

### Bacterial catalase activity

Extracellular bacterial catalase activity was measured by iodometric determination of H_2_O_2_ decomposed by bacterial cells as described in Ref. 35, with modifications. The assay was carried out as follows: 400 μL of cell suspension (or 400 μL of PBS in control probe) was mixed with 100 μL of 51 mm H_2_O_2_ solution in PBS and allowed to stand for 10 min at ambient temperature. For each *E. coli* sample, three dilutions in PBS (up to OD of 0.15, 0.30, and 0.60 at 540 nm) were used; the consumption of H_2_O_2_ by bacteria and the suspension optical density were directly proportional. Then, 20 μL of 6 N HCl and 500 μL of 245 mm solution of KI in H_2_O were added. Low pH provided the reaction of KI with H_2_O_2_ and inhibition of catalase activity. After 15 min of centrifugation at 600 ***g***, the OD of supernatants was read at 492 nm 35. The difference between the OD at 492 nm in the control (5 mm H_2_O_2_) and in the test probes was expressed in μmoles of H_2_O_2_ and normalized to the dry‐weight value.

### Fluorescence study of the hydrophobicity of cell envelope

The measurements of fluorescence (F) were performed according to Ref. 24 using a Hitachi F‐4000 fluorescence spectrophotometer and a 0.5 × 0.5 cm quartz cuvette. Fluorescence spectra of ANS (60 µm) and DMC (30 µm) were recorded at excitation wavelength of 360 nm and 420 nm, respectively. PBS or bacterial suspension was mixed with ANS or DMC before analysis; the final concentration of bacterial suspension was 0.8 ± 0.1 mg of DW·mL^−1^. The maximum fluorescence intensity was normalized to a dry‐weight basis.

To study the influence of the ionic strength of solution on the interaction between the fluorescent probes and bacteria, fluorescence intensity of the bound probe was measured twice in each sample: at the ionic strength of 0.157 m and 0.407 m. The ionic strength of the solution was increased by addition of 0.25 m NaCl to the mixture of the probes and bacterial cells in 0.01 m PBS. The ratio F(0.407)/F(0.157) was calculated, where F(0.157) and F(0.407) are the fluorescence intensity of the probe at the solution′s ionic strength of 0.157 m and 0.407 m, respectively 25. For negatively charged probe, ANS F(0.407)/F(0.157) >1 indicated negatively charged surface and F(0.407)/F(0.157) <1 suggested positively charged surface. DMC fluorescence was not expected to depend on the ionic strength of buffer solution since its molecule is uncharged.

### Microscopic study

Morphological changes in neutrophils in blood incubated with bacteria at a 1:6 ratio of neutrophils to bacteria were examined by light microscopy according to Ref. 36. About 3 μL of *E. coli* suspension in PBS (OD at 540 = 2.5) was mixed with 17 μL of a healthy volunteer’s blood. Incubation continued for 1 h at 37 °C with periodical shaking, and then, the smears were prepared. The smears were stained by the Romanowsky–Giemsa technique, and microscopic examination of stained cells was carried out using a Motic B3 light microscope.

### Statistics

The data are shown as a mean value ± standard deviation if not indicated otherwise. Correlations were calculated by the Pearson method; for nonlinear relations, Pearson’s approximation method was applied. The software statistica 6.0 was used for statistical analysis of data (Mann–Whitney *U*‐test or Student’s *t*‐test).

## Results

### CL analysis of neutrophil‐stimulating capacity and hydroperoxidase activity of *Escherichia coli*

**Figure 1:**
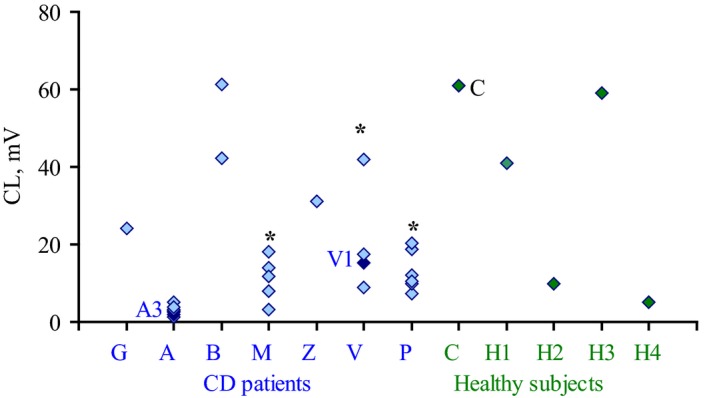
Chemiluminescence responses of neutrophils stimulated by *E. coli* isolates from CD (in blue) and healthy patients (in green) in whole blood of healthy volunteers. Each point represents CL maximum value for one *E. coli* isolate averaged for 3–5 independent experiments with 3–5 different blood samples. *Indicates patients with a significant difference between CL values of isolates within patient, *P* < 0.05 (Student’s *t*‐test).

Neutrophil CL was activated by bacterial suspensions in diluted blood of healthy volunteers, without leukocyte isolation 37 in order to imitate *in vivo* conditions. CL responses varied for different isolates from 2 to 70 mV (Fig. 1, Table 1). Isolates collected from the same patient differed in CL‐stimulating capacity by 1.5–5 times, and for some patients (M, V, and P), the difference was significant. The difference between isolates did not depend on blood sample used for analysis and was reproducible for at least three batches of each isolate. No significant difference in CL was detected between isolates from CD patients (23 isolates) vs healthy individuals (five isolates) (Fig. 1, Table 1).

**Figure 2:**
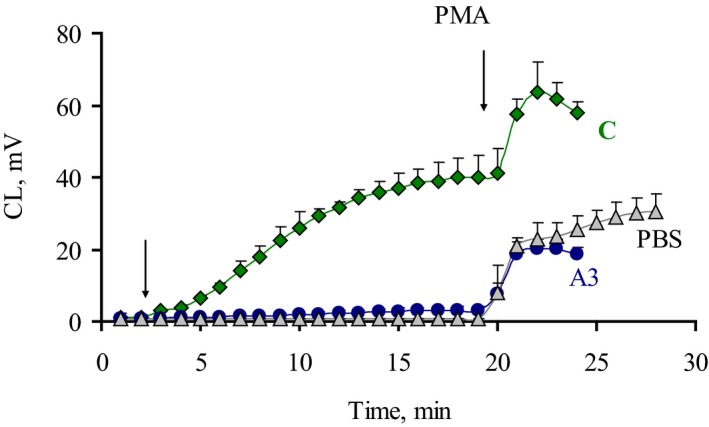
Time development and phorbol ester (PMA) stimulation effect on CL responses of blood neutrophils stimulated by *E. coli*. Typical time curves for PBS and isolates with low (A3) and high (C) CL values are shown, moments of stimulant addition indicated by arrows. Error bars represent SD.

Since bacterial toxins can influence neutrophil activity 38, isolates were tested for their ability to inhibit CL response induced in diluted blood. Phorbol ester (PMA) was added to the luminometer cuvette at the end of CL analysis, when the maximum value of CL induced by *E. coli* was achieved, and the second CL maximum was registered (Fig. 2). No significant effect of *E. coli* on subsequent PMA‐stimulated CL response was found, so the difference between the isolates could not result from any toxic effect of bacteria toward neutrophils.

**Figure 3:**
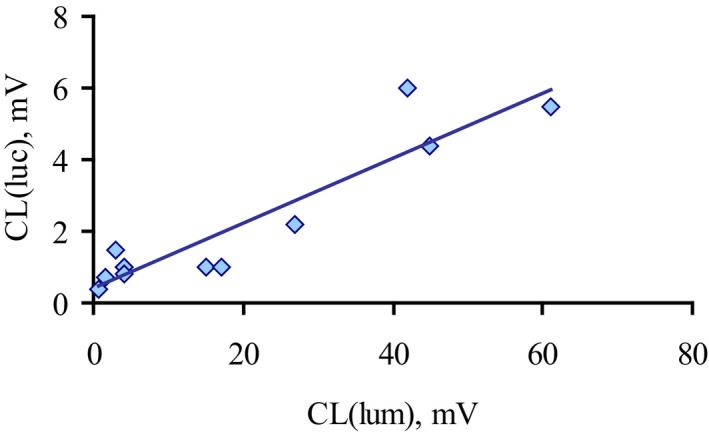
Correlation between luminol and lucigenin‐enhanced chemiluminescence of neutrophils stimulated by *E. coli* isolates from CD patients (*n* = 12). Luminol‐enhanced CL response of neutrophils in whole blood (CL (lum)) is plotted versus superoxide‐sensitive lucigenin‐enhanced CL (luc). Correlations were calculated by the Pearson method (*r* = 0.8, *P* < 0.05, *n* = 12).

Luminol‐enhanced CL stimulated in blood by *E. coli* isolates correlated with lucigenin‐enhanced CL, which is sensitive only to superoxide anion production (Fig. 3). Therefore, *E. coli* isolates that did not induce CL response in neutrophils probably managed it by disruption of superoxide produced by neutrophils.

In order to evaluate the ability of bacteria to protect themselves from ROS produced by neutrophils, catalase and peroxidase activities of *E. coli* isolates were assessed without cell destruction. Less than 10% of extracellular peroxidase activity was solubilized by Triton X‐100, indicating that the major part of enzymes was cell wall‐bound (tested for 12 *E. coli* isolates, data not shown).

**Figure 4:**
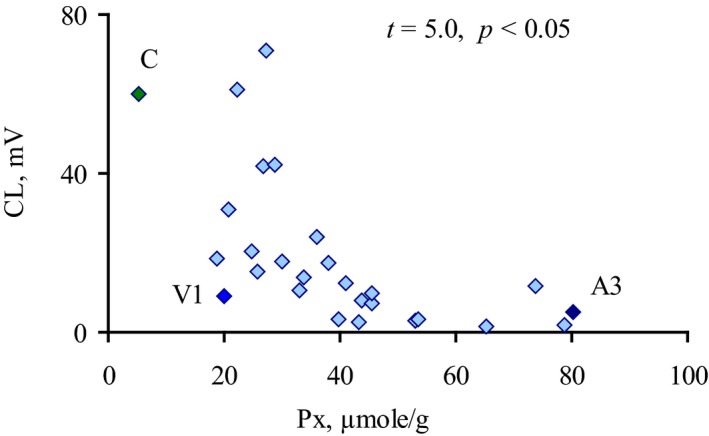
Correlation between CL response of whole blood neutrophils to *E. coli* isolates and their peroxidase activity. Peroxidase activity (Px) is given in µmol of oxidized o‐dianisidine per g of bacterial pellet dry weight. Significance (*P*) was quantified by the Pearson method for nonlinear correlation (*t*_0.5_ = 2.05). Values for isolates C, A3, and V1 (analyzed in microscopic assay with neutrophils below) are indicated by black diamonds.

We found that *E. coli* isolates with the highest peroxidase activity induced low CL response, while the isolates with significant CL‐stimulating capacity showed the lowest peroxidase activity, and this negative correlation was significant (*P* < 0.05; Fig. 4). There was no isolate combining high peroxidase‐ and CL‐stimulating activities. Bacterial catalase activity showed the same tendency, but correlation between catalase activity and CL values was not significant.

Since luminol‐enhanced CL depends on H_2_O_2_ and H_2_O_2_‐derived HOCl 9, the direct effect of peroxidase activity on CL should be evaluated. Exogenous peroxidase (HRP) was added to the CL probes with isolate C (with the lowest peroxidase activity of its own; Table 1) so that its activity was equal to the highest demonstrated by *E. coli* isolates in the current study (86.4 ± 3.3 µmol·g^−1^ dry weight; Table 1). This did not affect CL response of blood neutrophils, suggesting that the low CL response is not due to chemiluminescence inhibition by ROS eliminating.

### Morphological analysis of neutrophil activation

Microscopic study of morphological alterations of stimulated neutrophils was performed to detect whether there are other signs of activation induced in neutrophils by *E. coli* isolates with low CL‐inducing activity. As such, two CD isolates with low CL activity were selected—A3 and V1. Isolate C (from healthy patient) was selected as a control isolate with the one of the highest neutrophil‐stimulating activities (regarding CL response induction).

**Figure 5:**
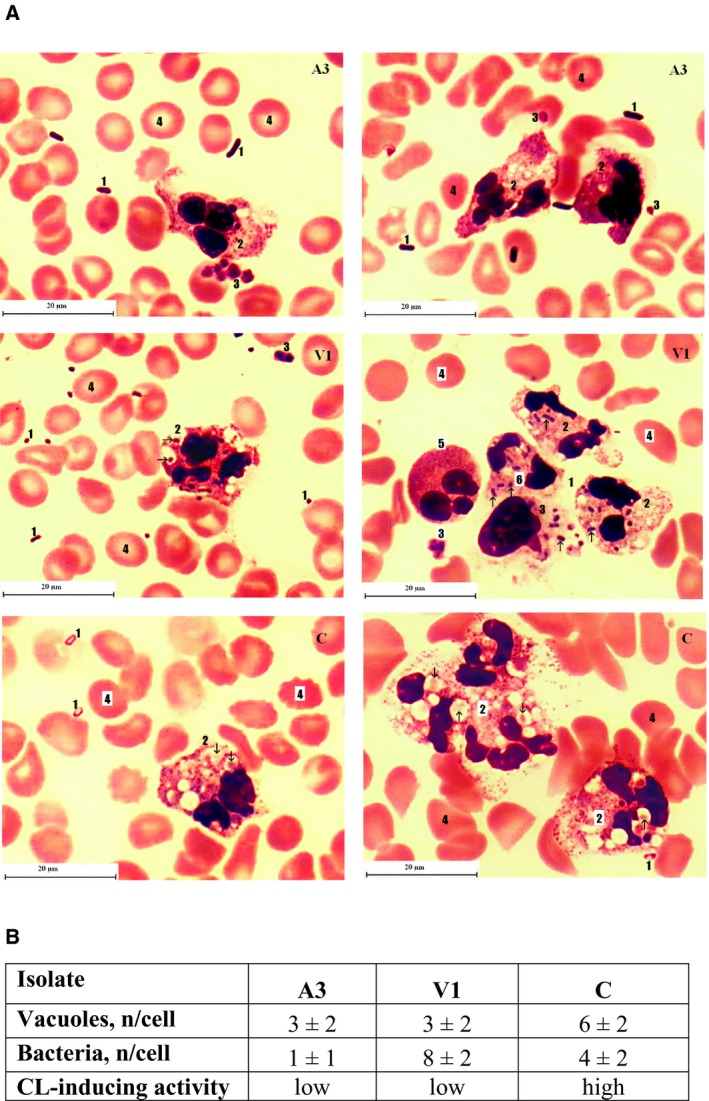
Effect of *E. coli* isolates with diverse CL‐stimulating activity on the morphological features of neutrophils. Whole blood samples were incubated with *E. coli* isolates that have induced low (A3 and V1) and high (C) CL response. The Romanowsky–Giemsa staining was used to visualize the details. At least 50 microscope fields of view per isolate were evaluated for picture and calculations. (A) Typical picture of activated neutrophil for each isolate: 1—*E. coli* cells; 2—neutrophils/neutrophil aggregates containing vacuoles (arrows indicate the captured bacterial cells); 3—platelets, 4—erythrocytes; 5—eosinophil; and 6—monocyte with captured bacteria. The length of the scale bars is 20 μm. (B) Parameters of phagocytic activity of neutrophils stimulated by *E. coli* isolates. Mean values are given ± SD.

Figure 5 demonstrates that all isolates induced signs of neutrophil activation: swelling of neutrophils nuclei, formation of vacuoles, and cell aggregation (Fig. 5A).

Isolate with the lowest CL‐stimulating activity (A3) was the least susceptible to the phagocytosis (Fig. 5B), even though the neutrophils incubated with it showed signs of activation. Only the cells of isolate with the highest CL‐stimulating activity (C) showed signs of degradation in phagolysosome (the engulfed bacterial cells differ in form and staining from the free ones). This isolate also induced higher number of vacuoles formed within neutrophil cells. As for the isolate V, the vacuoles with bacteria were rather phagosomes, smaller than for isolate C, and with no signs of bacterial digestion.

### Fluorescent analysis of hydrophobicity of *Escherichia coli *cell surface

As shown by the experiments above, the difference in neutrophil capturing capacity toward *E. coli* could not be entirely attributed to the activity of bacterial hydrogenases or to the direct inhibiting effect of bacteria on neutrophils. Another factor contributing in that is the possible difference in the cell surface properties of various isolates.

*Escherichia coli* cell surface properties were studied by fluorescent probes ANS (negatively charged amphiphilic) and DMC (neutral hydrophobic). Addition of bacterial suspensions significantly increased probes’ fluorescence intensity: from 2.4 ± 0.3 to 16–64 a.u. for ANS and from 1.4 ± 0.1 to 2 – 110 a.u. for DMC. The averaged values for isolates within patients are given in Table 1.

Binding of the probes to the bacterial surface and transition from an aquatic to a hydrophobic environment were proved by the blue shift of the fluorescence maxima 39, 40, 41 registered for ANS (from 512 ± 1 to 490 ± 2nm) and for DMC (from 522 ± 1 nm to 499 ± 1 nm) in bacterial suspensions.

**Figure 6:**
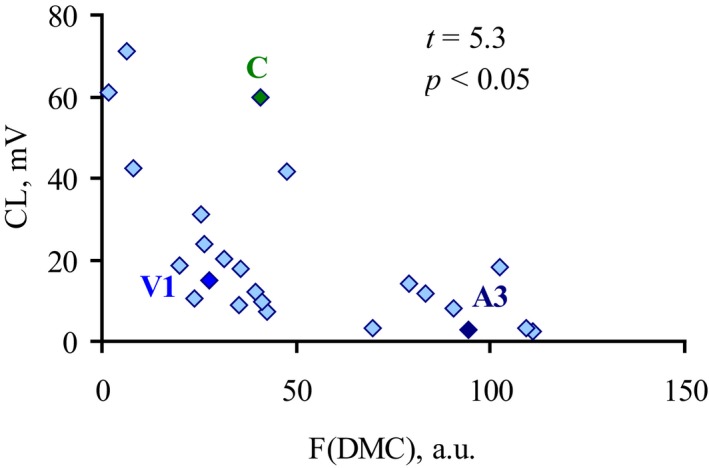
Correlation between CL response of whole blood neutrophils induced by *E. coli* isolates and their DMC fluorescence intensity reflecting bacterial surface hydrophobicity. DMC hydrophobic fluorescent probe was added to 24 isolate bacterial suspensions (Table 1), and F(DMC) was recorded at 499 nm. Significance (*P*) was quantified by the Pearson method for nonlinear correlation (*t*_0.5_ = 2.05).

Fluorescence intensity of both probes negatively correlated with CL values: *r *= −0.46 in the experiments with ANS (data not shown) and *r *= −0.63 in the experiments with DMC, where the correlation was significant at *P* < 0.05 (Fig. 6).

To establish the role of electrostatic forces in the interaction of the probes with *E. coli*, the effect of the increase in ionic strength was studied. We detected approximately 10% elevation of ANS fluorescence intensity, F(0.407)/F(0.157) = 1.07 ± 0.08. Thus, the contribution of electrostatic forces to the binding of the probe to *E. coli* was not significant. Fluorescence values in experiments with DMC were not influenced by ionic forces and therefore resulted from hydrophobic binding of the probe to the bacterial cell surface.

### Analysis of the adhesive and invasive ability of *Escherichia coli* isolates

Analysis of the ability of *E. coli* isolates to adhere and invade CaCo cells was performed for eight isolates from seven CD patients that demonstrated high, low, and intermediate values of neutrophil‐stimulating peroxidase and catalase activities (Table 1), two isolates from healthy individuals, and one laboratory strain MG1655 (Table 2). Higher adhesion ability (5–30 times more than the laboratory strain) was demonstrated by four isolates (V2, V4, M4, and G). Invasion was demonstrated by three isolates (V2, M4, and P2). Therefore, adhesive–invasive activity was observed for two isolates (V2 and M4).

**Table 2:** Adhesion and invasion assay

	V2	V4	M4	G	P2	A1	B1	H1	C	MG1655
CFU per agar plate in control ×10^4^	19.5 ± 1.92	22.87 ± 2.23	18.13 ± 2.03	13.25 ± 1.91	55.67 ± 4.59	74.67 ± 4.03	76.17 ± 4.07	46.83 ± 4.07	50 ± 4	18.875 ± 2.53
CFU per agar plate after adhesion. ×10^3^	9.75 ± 1.83	18.37 ± 6.57	59.33 ± 9.09	11 ± 0.93	3.67 ± 0.82	3.5 ± 0.55	8.83 ± 1.47	1.5 ± 0.55	4.33 ± 0.82	1.9875 ± 0.23
CFU per agar plate after gentamicin treatment	12.5 ± 4.63	0	47.5 ± 11.65	0	13.33 ± 5.16	0	41.67 ± 9.83	0	0	0
Number of adhered–invaded bacteria per CaCo cell	0.02	0.04	0.12	0.02	0.007	0.007	0.018	0.003	0.009	0.004
% of adhered–invaded bacteria in bacterial suspension	5	8.03	32.74	8.3	0.66	0.47	1.16	0.32	0.87	1.05
% of invaded bacteria	0.13	0	0.08	0	0.36	0	0.47	0	0	0

Values are given ± SD (standard deviation).

No correlation was observed between adhesive and invasive ability of *E. coli* isolates and the intensity of neutrophil stimulation (Tables 1 and 2). Isolates with the lowest CL values (A1) and with the highest (C) demonstrated no adhesion and invasion activity. Isolates with maximum observed adhesive–invasive isolates demonstrated both high (V2) and low CL values (M4).

### *Escherichia coli* isolates fall into three groups

The isolates could be divided into three groups on the basis of their CL‐stimulating capacity: group 1 with CL values of 1−10 mV, group 2 with CL values of 11–30 mV, and group 3 with CL of more than 31 mV (Table 1).

**Figure 7:**
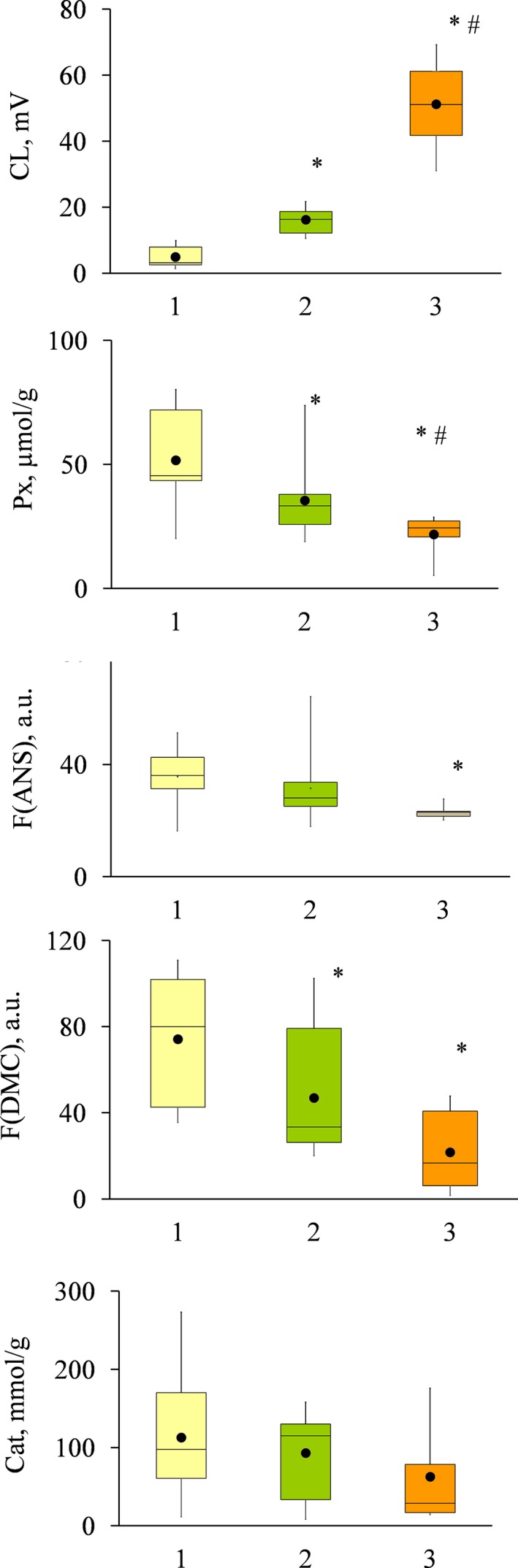
Three groups of *E. coli* isolates. *E. coli* isolates were arranged in three groups according to the CL‐inducing activity (from the lowest to the highest, 1, 2, and 3). The following parameters were compared within/between the groups: extracellular peroxidase activity (Px, μmol·g^−1^ DW), cell surface hydrophobicity defined by probe fluorescence (F(ANS), a. u., and F(DMC), a.u.), and extracellular catalase activity (catalase, nmol·g^−1^). Significant difference is indicated: *—in comparison with group 1; #—in comparison with group 2 (*P* < 0.05, Mann–Whitney test).

As Fig. 7 demonstrates, cell surface hydrophobicity (F(DMC)) and peroxidase activity (Px) significantly differed between these groups.

These data suggest the existence of three different *E. coli* phenotypes differing in their ability to evade ROS production by neutrophils, extracellular peroxidase activity, and cell surface hydrophobicity. *Escherichia coli* isolates of the same origin can belong to different phenotypes (patients ‘M’, ‘V’, and ‘P’), or to one (patient ‘A’ and ‘B’).

Parameters such as the source of isolate, the severity of disease, inflammation localization, and sex/age of patients and isolates phylogroup showed no correlation with the intensity of neutrophil stimulation or bacterial hydroperoxidase activity (Table 1, File S1).

## Discussion

The role of *E. coli* in the acute inflammation during Crohn’s disease is still disputable 42, 43, 44. However, multiple observations of *E. coli* intensive growth in the afflicted intestine suggest that bacteria proliferating under such conditions have to develop some mechanisms of resistance to ROS/RHS or avoiding their formation. The aim of the current study was to evaluate various isolates of *E. coli* in regard to their interaction with inflammation agents, such as neutrophils and ROS.

We have observed high variability in these parameters in *E. coli* from CD patients. In particular, a phenotype was detected combining low neutrophil activation with high ROS utilization activity. About 39% of the total number of *E. coli* isolates from CD patients (9 isolates of 23) induced CL response below 10 mV, and four of seven CD patients involved into the study had in their intestine at least one isolate with low CL‐stimulating activity. At the same time, 17.4% of CD isolates (4 out of 23) induced high CL responses and showed much lower peroxidase activity.

The low CL‐inducing isolates were not completely ‘invisible’ to neutrophils, as was shown by microscopic study in the experiments with the isolates A3 and V1.

It is necessary to mention that low CL values were indeed correct indications of decreased neutrophil activation, and not a result of bacterial utilization of ROS produced by activated neutrophils. This was confirmed by the fact that luminol CL values correlated with lucigenin CL independent of H_2_O_2_ production by neutrophils, and was insensitive to exogenous peroxidase (HRP 45). Presumably, isolates with low CL‐stimulating capacity were able to avoid the induction of ROS/RHS production at all during neutrophil activation.

Previously published researches suggested that bacterial cell surface hydrophobicity could influence the scenario of neutrophil activation, and bacteria with high hydrophobicity should have the most stimulating capacity 17, 18. Unexpectedly, in the studied *E. coli* isolates, the higher hydrophobicity corresponded to the lower CL values and the higher peroxidase activity.

Alignment of *E. coli* genome sequences from different intestine parts of one patient performed earlier 29 revealed only minor heterogeneity and some deletions (usually a deletion of one of the smaller contigs, probably a plasmid). At the same time, sequences of genes identified as encoding catalases and peroxidases of isolates from one patient were identical, since none of the found polymorphisms was located in those genes. Therefore, one may suggest that the difference in phenotype of genetically close isolates from one patient observed in the current study (patients V, M, and P) could be due to the mobile elements of genome that could be achieved via horizontal transfer.

Hence, we report that *E. coli* isolates from CD patients are rather variable regarding the interaction with inflammation agents. One of the phenotypes observed combines the ability for low neutrophil activation with comparatively high ability to utilize extracellular ROS and increased cell surface hydrophobicity.

## Author contributions

MAM, TVV, JPB, LJB, SAG, AAG, NVS, and GED performed the laboratory section of the study. EVM analyzed the data. PLS, AIP, and NAF performed the clinic part of the study. EVM, DVR, OMP, OVP, and VMG were involved in study designing, writing, and editing of the manuscript. All authors read and approved the final manuscript.

## Conflict of interest

The authors declare no conflict of interest.

## Supporting information


**File S1.** Parameters of *E. coli* isolates are presented on 3D plot: CL (Chemiluminescent responce intensity, lum), Cat (extracellular catalase activity, nmol H_2_O_2_/ g dry weight) and Px (peroxidase activity, µmol of oxidized o‐dianisidine per g of bacterial pellet dry weight).

## References

[1] Conte MP , Schippa S , Zamboni I , Penta M , Chiarini F , Seganti L , Osborn J , Falconieri P , Borrelli O and Cucchiara S (2006) Gut‐Associated bacterial microbiota in paediatric patients with inflammatory bowel disease. Gut 55, 1760–1767. 16648155 10.1136/gut.2005.078824 PMC1856471

[2] Gevers D , Kugathasan S , Denson LA , Vazquez‐Baeza Y , Van Treuren W , Ren B , Schwager E , Knights D , Song SJ , Yassour M *et al* (2014) The treatment‐naive microbiome in new‐onset Crohn’s disease. Cell Host Microbe 15, 382–392. 24629344 10.1016/j.chom.2014.02.005 PMC4059512

[3] Yang Y and Jobin C (2014) Microbial imbalance and intestinal pathologies: connections and contributions. Dis Mod Mech 7, 1131–1142. 10.1242/dmm.016428 PMC4174524 25256712

[4] Carrière J , Darfeuille‐Michaud A and Nguyen HT (2014) Infectious etiopathogenesis of Crohn’s disease. World J Gastroenterol 20, 12102–12117. 25232246 10.3748/wjg.v20.i34.12102 PMC4161797

[5] Demirel I , Kinnunen A , Önnberg A , Söderquist B and Persson K (2013) Comparison of host response mechanisms evoked by extended spectrum beta lactamase (ESBL)‐ and non‐ESBL‐producing uropathogenic *E. coli* . BMC Microbiol 31, 181. 10.1186/1471-2180-13-181 PMC3733941 24059789

[6] Mouzaoui S , Djerdjouri B , Makhezer N , Kroviarski Y , El‐Benna J and Dang PM (2014) Tumor necrosis factor‐α‐induced colitis increases NADH oxidase 1 expression, oxidative stress, and neutrophil recruitment in the colon: preventive effect of apocynin. Mediat Inflamm 2014, 312484. 10.1155/2014/312484 PMC4167951 25276054

[7] Alzoghaibi M (2013) Concepts of oxidative stress and antioxidant defense in Crohn’s disease. World J Gastroenterol 19, 6540–6547. 24151379 10.3748/wjg.v19.i39.6540 PMC3801366

[8] Kruidenier L , Kuiper I , Lamers CB and Verspaget HW (2003) Intestinal oxidative damage in inflammatory bowel disease: semi‐quantification, localization, and association with mucosal antioxidants. J Pathol 201, 28–36. 12950014 10.1002/path.1409

[9] Vladimirov YA and Proskurnina EV (2009) Free radicals and cell chemiluminescence. Biochemistry (Moscow) 74, 1545–1566. 20210708 10.1134/s0006297909130082

[10] Visick JE and Clarke S (1997) RpoS‐ and OxyR‐independent induction of HPI catalase at stationary phase in *Escherichia coli* and identification of rpoS mutations in common laboratory strains. J Bacteriol 179, 4158–4163. 9209028 10.1128/jb.179.13.4158-4163.1997 PMC179234

[11] Shiratsuchi A , Shimamoto N , Nitta M , Tuan TQ , Firdausi A , Gawasawa M , Yamamoto K , Ishihama A and Nakanishi Y (2014) Role for s38 in prolonged survival of *Escherichia coli* in *Drosophila melanogaster* . J Immunol 192, 666–675. 24337747 10.4049/jimmunol.1300968

[12] Farr SB and Kogoma T (1991) Oxidative stress responses in *Escherichia coli* and *Salmonella typhimurium* . Microbiol Rev 55, 561–585. 1779927 10.1128/mr.55.4.561-585.1991 PMC372838

[13] Loewen PC , Switala J and Triggs‐Raine BL (1985) Catalases HPI and HPII in *Escherichia coli* are induced independently. Arch Biochem Biophys 243, 144–149. 3904630 10.1016/0003-9861(85)90782-9

[14] Claiborne A and Fridovich I (1979) Purification of the o‐dianisidine peroxidase from *Escherichia coli* B. Physicochemical characterization and analysis of its dual catalatic and peroxidatic activities. J Biol Chem 254, 4245–4252. 374409

[15] Loewen P (1996) Probing the structure of catalase HPII of *Escherichia coli –* a review. Gene 179, 39–44. 8955627 10.1016/s0378-1119(96)00321-6

[16] Alteri CJ , Lindner JR , Reiss DJ , Smith SN and Mobley HL (2011) The broadly conserved regulator PhoP links pathogen virulence and membrane potential in *Escherichia coli* . Mol Microbiol 82, 145–163. 21854465 10.1111/j.1365-2958.2011.07804.x PMC3188958

[17] Magnusson KE , Davies J , Grundström T , Kihlström E and Normark S (1980) Surface charge and hydrophobicity of *Salmonella*, *E. coli*, *Gonococci* in relation to their tendency to associate with animal cells. Scand J Infect Dis Suppl 24, 135–140. 6782659

[18] Williams P , Lambert PA , Haigh CG and Brown MR (1986) The influence of the O and K antigens of *Klebsiella aerogenes* on surface hydrophobicity and susceptibility to phagocytosis and antimicrobial agents. J Med Microbiol 21, 125–132. 2419562 10.1099/00222615-21-2-125

[19] Lock R , Dahlgren C , Linden M , Stendahl O , Svensbergh A and Ohman L (1990) Neutrophil killing of two type 1 fimbria‐bearing *Escherichia coli* strains: dependence on respiratory burst activation. Infect Immun 58, 37–42. 1967171 10.1128/iai.58.1.37-42.1990 PMC258405

[20] Blomgran R , Zheng L and Stendahl O (2004) Uropathogenic *Escherichia coli* triggers oxygen‐dependent apoptosis in human neutrophils through the cooperative effect of type 1 fimbriae and lipopolysaccharide. Infect Immun 72, 4570–4578. 15271917 10.1128/IAI.72.8.4570-4578.2004 PMC470702

[21] Dreux N , Denizot J , Martinez‐Medina M , Mellmann A , Billig M , Kisiela D , Chattopadhyay S , Sokurenko E , Neut C , Gower‐Rousseau C *et al* (2013) Point mutations in FimH adhesin of Crohn’s disease‐associated adherent‐invasive *Escherichia coli* enhance intestinal inflammatory response. PLoS Pathog 9, e1003141. 23358328 10.1371/journal.ppat.1003141 PMC3554634

[22] Park BS and Lee JO (2013) Recognition of lipopolysaccharide pattern by TLR4 complexes. Exp Mol Med 45, e66. 24310172 10.1038/emm.2013.97 PMC3880462

[23] Nivea‐Gomez D and Gennis RB (1997) Affinity of intact *Escherichia coli* for hydrophobic membrane probes is a function of the physiological state of the cells. Proc Natl Acad Sci USA 74, 1811–1815. 10.1073/pnas.74.5.1811 PMC431011 325554

[24] Phillips SK and Cramer WA (1973) Properties of the fluorescence probe response associated with the transmission mechanism of colicin E1. Biochemistry 12, 1170–1176. 4569773 10.1021/bi00730a024

[25] Dobretsov GE , Gularyan SK , Panasenko OM , Melnichenko AA and Orekhov AN (2006) The charge of the surface layer of multiply modified low‐density lipoproteins. Biochem (Moscow), Series A Memb Cell Biol 23, 420–425.

[26] Mikhalchik E , Balabushevich N , Vakhrusheva T , Sokolov A , Baykova J , Rakitina D , Shcherbakov P , Gusev S , Gusev A , Kharaeva Z *et al*, (2019) Mucin adsorbed by *E. coli* can affect neutrophil activation in vitro. FEBS Open Bio 10, 180–196.. 10.1002/2211-5463.12770 PMC6996330 31785127

[27] Beers RF and Sizer IW (1952) A spectrophotometric method for measuring the breakdown of hydrogen peroxide by catalase. J Biol Chem 195, 133–140. 14938361

[28] Porebski S , Bailey GL and Baum BR (1997) Modification of a CTAB DNA extraction protocol for plants containing high polysaccharide and polyphenol components. Plant Mol Biol Rep 15, 8–15.

[29] Rakitina DV , Manolov AI , Kanygina AV , Garushyants SK , Baikova JP , Alexeev DG , Ladygina VG , Kostryukova ES , Larin AK , Semashko TA *et al* (2017) Genome analysis of *E. coli* isolated from Crohn's disease patients. BMC Genom 18, 544. 10.1186/s12864-017-3917-x PMC5517970 28724357

[30] Silverberg MS , Satsangi J , Ahmad T , Arnott ID , Bernstein CN , Brant SR , Caprilli R , Colombel J‐F , Gasche C , Geboes K *et al* (2005) Toward an integrated clinical, molecular and serological classification of inflammatory bowel disease: report of a working party of the 2005 Montreal World Congress of Gastroenterology. Can J Gastroenterol 19 (supplA), 5–36. 10.1155/2005/269076 16151544

[31] Best WR , Becktel JM , Singleton JW and Kern F Jr (1976) Development of a Crohn's disease activity index. Nat Cooperat Crohn's Dis Study Gastroenterol 70, 439–444. 1248701

[32] Kondratyeva K , Wollman A , Gerlitz G and Navon‐Venezia S (2017) Adhesion and invasion to epithelial cells and motility of extended‐spectrum b‐lactamase‐producing *Escherichia coli* reveal ST131 superiority: a comparative in vitro study of extraintestinal pathogenic *E. coli* lineages. J Med Microbiol 66, 1350–1357. 28825894 10.1099/jmm.0.000549

[33] Easmon CSF , Cole PJ , Williams AJ and Hastings M (1980) The measurement of opsonic and phagocytic function by Luminol‐dependent chemiluminescence. Immunology 41, 67–74. 7429554 PMC1458226

[34] Andrews PC , Parens C and Krinsky NI (1984) Comparison of myeloperoxidase and hemi‐myeloperoxidase with respect to catalysis, regulation and bactericidal activity. Arch Biochem Biophys 228, 439–442. 6320739 10.1016/0003-9861(84)90008-0

[35] Bukharin OV , Cherkasov SV , Sgibnev AV , Zabirova TM and Ivanov YB (2000) Effects of microbial metabolites on catalase activity and growth of *Staphylococcus aureus* 6538P. Bull Exp Biol Med 130, 679–681. 11140585 10.1007/BF02682104

[36] Brest P , Bétis F , Çuburu N , Selva E , Herrant M , Servin A , Auberger P and Hofman P (2004) Increased rate of apoptosis and diminished phagocytic ability of human neutrophils infected with Afa/Dr diffusely adhering *Escherichia coli* strains. Infect Immun 72, 5741–5749. 15385473 10.1128/IAI.72.10.5741-5749.2004 PMC517549

[37] Lindena J , Burkhardt H and Dwenger A (1987) Mechanisms of non‐opsonized zymosan‐induced and luminol‐enhanced chemiluminescence in whole blood and isolated phagocytes. J Clin Chem Clin Biochem 11, 765–778. 10.1515/cclm.1987.25.11.765 3440857

[38] Russo TA , Davidson BA , Genagon SA , Warholic NM , Macdonald U , Pawlicki PD , Beanan JM , Olson R , Holm BA and Knight PR 3rd (2005) *E. coli* virulence factor hemolysin induces neutrophil apoptosis and necrosis/lysis in vitro and necrosis/lysis and lung injury in a rat pneumonia model. Am J Physiol Lung Cell Mol Physiol 289, L207–L216. 15805136 10.1152/ajplung.00482.2004

[39] Stryer L (1965) The interaction of a naphthalene dye with apomyoglobin and apohemoglobin. A fluorescent probe of non‐polar binding sites. J Mol Biol 13, 482–495. 5867031 10.1016/s0022-2836(65)80111-5

[40] Dobretsov GE , Petrov VA , Mishijev VE , Klebanov GI and Vladimirov YuA (1977) 4‐Dimethylaminochalcone and 3‐methoxybenzanthrone as fluorescent probes to study biomembranes. I. Spectral characteristics. Stud Biophys 65, 91–98.

[41] Bakhshiev NG , Gularyan SK , Dobretsov GE , Kirillova AYu and Svetlichnyi VYu (2006) Solvatochromism and solvatofluorochromism of the intramolecular charge transfer band of 4‐dimethylaminochalcone in the electronic spectra of its solutions. Opt Spectrosc 100, 700–708.

[42] Strober W (2011) Adherent‐invasive *E. coli* in Crohn disease: bacterial “agent provocateur”. J Clin Invest 121, 841–844. 21339637 10.1172/JCI46333 PMC3049399

[43] Agus A , Massier S , Darfeuille‐Michaud A , Billard E and Barnich N (2014) Understanding host‐adherent‐invasive *Escherichia coli* interaction in Crohn's disease: opening up new therapeutic strategies. Biomed Res Int 2014, 567929. 25580435 10.1155/2014/567929 PMC4279263

[44] Martinez‐Medina M and Garcia‐Gil LJ (2014) *Escherichia coli* in chronic inflammatory bowel diseases: An update on adherent invasive *Escherichia coli* pathogenicity. World J Gastrointest Pathophysiol 5, 213–227. 25133024 10.4291/wjgp.v5.i3.213 PMC4133521

[45] Kopprasch S , Kohl M and Graessler J (1998) Different effects of exogenous horseradish peroxidase on luminol‐amplified chemiluminescence induced by soluble and particulate stimuli in human neutrophils. J Biolumin Chemilumin 13, 267–271. 9839190 10.1002/(SICI)1099-1271(1998090)13:5<267::AID-BIO496>3.0.CO;2-K

