# Supplementary material for: Neutrophil activation by Escherichia coli isolates from human intestine: effects of bacterial hydroperoxidase activity and surface hydrophobicity
Source: FEBS Open Bio. 2020 Feb 5;10(3):414–26. doi: 10.1002/2211-5463.12796 (PMC7050253; doi:10.1002/2211-5463.12796)

**Supplementary data file 1**

Parameters of *E. coli* isolates are presented on 3D plot: CL (Chemiluminescent responce intensity, lum), Cat (extracellular catalase activity, nmol H_2_O_2_/ g dry weight) and Px (peroxidase activity, µmol of oxidized o-dianisidine per g of bacterial pellet dry weight). Dots color indicates other parameters (patient, clinical or endoscopic disease activity, lesions localization, *E. coli* sample source, CL response groups (1, 2, 3).


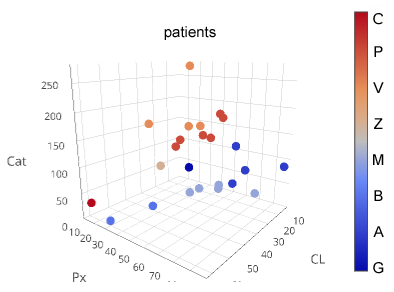


**Clinical activity of CD**


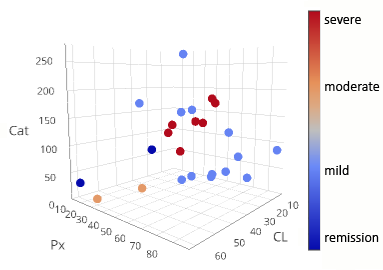


**Endoscopic activity of CD**


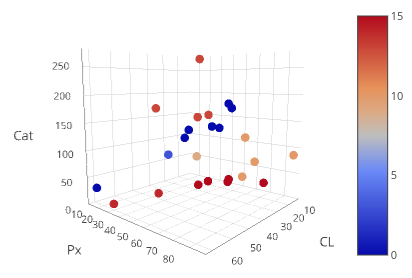


**Sex (plot in two projections)**


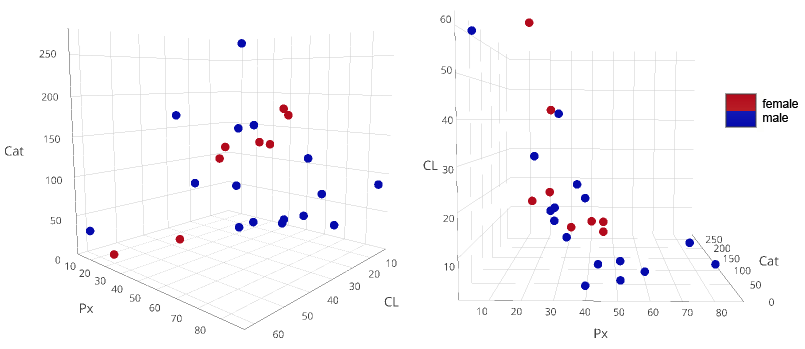


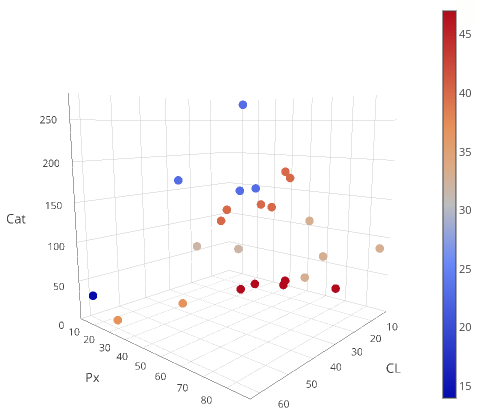


age


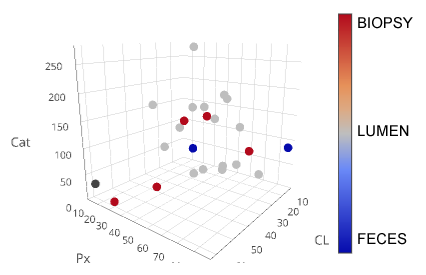


source of *E. coli* samples


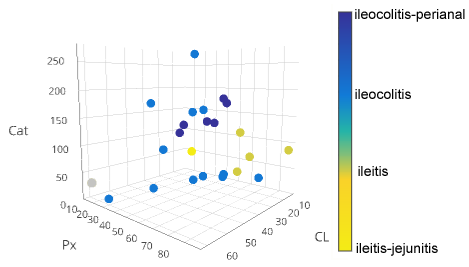


localization of lesions


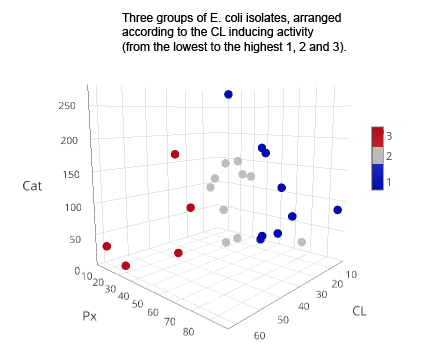

Supplement: Supplementary file 1 — File S1. Parameters of E. coli isolates are presented on 3D plot: CL (Chemiluminescent responce intensity, lum), Cat (extracellular catalase activity, nmol H2O2/ g dry weight) and Px (peroxidase activity, µmol of oxidized o‐dianisidine per g of bacterial pellet dry weight). [file FEB4-10-414-s001.doc]
